# The Association Between Tea Consumption Timing and Kidney Function: Insights From a National Survey

**DOI:** 10.1002/fsn3.70787

**Published:** 2025-09-26

**Authors:** Xuetong Tang, Jiankui Guo, Wen Hu, Yuan Liu, Yunying Shi

**Affiliations:** ^1^ West China School of Public Health Sichuan University Chengdu Sichuan China; ^2^ Department of Clinical Nutrition, West China Hospital Sichuan University Chengdu Sichuan China; ^3^ Department of Nephrology, West China Hospital Sichuan University Chengdu Sichuan China

**Keywords:** chronic kidney disease (CKD), circadian rhythm, estimated glomerular filtration rate (eGFR), NHANES database, tea consumption

## Abstract

Tea consumption has garnered significant attention due to its rich polyphenolic compounds and associated health benefits. However, existing research primarily focuses on the quantity of tea intake, while studies examining tea consumption timing remain limited. Given the circadian rhythm of kidney function and its metabolic implications, this study investigated the association between tea consumption timing, estimated glomerular filtration rate (eGFR), and chronic kidney disease (CKD) risk. Based on NHANES data from 2005 to 2018, 40,496 participants were included. Participants were grouped into high, medium, low, and non‐tea intake groups. Additionally, tea consumption timing was categorized into four periods: dawn to noon, noon to afternoon, afternoon to night, and night to dawn. Multivariate regression models and restricted cubic spline models were employed to evaluate the associations between tea consumption timing, intake levels, and eGFR. Tea consumption timing showed a significant time‐dependent association with eGFR levels. After adjusting for confounders, dawn‐to‐noon tea consumption demonstrated the strongest positive association with higher eGFR (3.18 mL/min/1.73 m^2^, *p* < 0.001), followed by noon to afternoon (1.12, *p* < 0.001) and afternoon to night (0.71, *p* < 0.01). Stratified analyses revealed that daytime tea consumption was significantly associated with higher eGFR levels in middle‐aged and older groups, whereas nighttime tea consumption in the younger group showed a slight adverse association. Analyses by tea type showed consistently positive associations for black tea, while green and other teas showed more variable results. This study underscores the significant association of tea consumption timing on kidney function, with the strongest associations observed during the dawn‐to‐noon period. Given the cross‐sectional design of this study, only associations were identified; thus, future longitudinal studies are necessary to confirm these findings. Adjusting tea timing may be a potential dietary consideration for CKD prevention, but further studies are warranted.

## Graphical abstract

This study investigates the association between tea consumption timing and kidney function, using data from 40,496 participants. We found that tea consumption, particularly in the dawn‐to‐noon period, is positively associated with better estimated glomerular filtration rate and reduced risk of chronic kidney disease. These findings highlight the importance of tea timing in promoting kidney health.

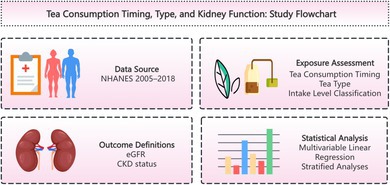

## 1 Introduction

Chronic kidney disease (CKD) is a progressive condition characterized by a sustained decline in glomerular filtration rate (GFR) lasting 3 months or longer (Cockwell and Fisher 2020). The global prevalence of CKD has exceeded 10% and continues to rise (Kövesdy 2022). From 1990 to 2016, the global burden of CKD increased by 87% (Jha and Modi 2018). This trend is particularly pronounced among older populations, where the incidence of CKD has significantly risen, posing profound challenges to both individual health and public health systems. As the prevalence of CKD continues to grow, the demand for renal replacement therapies, such as dialysis and transplantation, is expected to increase markedly, imposing a substantial economic burden on health care systems worldwide (Pereira‐Morales and Rojas 2022; Neuen et al. 2023). Therefore, the prevention and management of CKD represent not only a medical challenge but also a critical global public health priority.

Tea is a widely consumed beverage worldwide, and its health effects have attracted considerable attention in recent years. Rich in active compounds such as polyphenols (e.g., catechins), caffeine, and amino acids, tea not only provides a distinctive flavor profile but also offers potential health benefits through various biological mechanisms (Khan and Mukhtar 2018; Ju et al. 2007). Among these compounds, polyphenols represent one of the primary chemical constituents of tea, accounting for 30%–40% of its dry weight (Lin et al. 2003). Renowned for their antioxidant, anti‐inflammatory, and anticarcinogenic properties, polyphenols have been shown to be associated with improvements in kidney health in patients with CKD. Research indicates that the antioxidant properties of polyphenols can alleviate oxidative stress in the kidneys, which may be associated with improved renal function (Liczbiński and Bukowska 2022; Rodrigo and Bosco 2006). Several studies have established a correlation between increased tea consumption and a reduced risk of CKD (Liu, Zhang, et al. 2023; Liu, Yang, et al. 2023). Furthermore, higher tea intake has been associated with improvements in renal tubular function, including estimated glomerular filtration rate (eGFR), as well as reductions in urinary albumin levels (Zhang et al. 2022).

While the potential health benefits of tea consumption have been preliminarily established, existing research has primarily concentrated on the relationship between the quantity of tea intake and health outcomes. There is a notable lack of studies investigating the differential associations of the timing of tea consumption on health. The timing of tea consumption may be associated with differences in the absorption and metabolism of its constituents, which may be associated with different physiological responses. For instance, consuming tea in the morning may be associated with increased metabolic activity and antioxidant status, whereas drinking tea at night may be associated with poorer sleep quality and potential disruptions in kidney detoxification rhythms (Albrecht 2011; Burke et al. 2015; Oike et al. 2014). In individuals with CKD, the circadian rhythm of kidney function and the potential regulatory roles of tea on renal metabolism remain insufficiently explored. Understanding the relationship between the timing of tea consumption and changes in kidney function could not only optimize tea‐drinking habits but also may provide insights for personalized dietary considerations for CKD patients.

This study aims to analyze the overall association between tea consumption and CKD risk using data from the National Health and Nutrition Examination Survey (NHANES). Furthermore, it investigates the differential associations of tea consumption timing on renal function indicators, which may inform potential dietary strategies for the prevention and management of CKD.

## 2 Methods

### 2.1 Study Design and Data Source

The NHANES is a comprehensive nationwide health study conducted by the National Center for Health Statistics (NCHS) in the United States. It employs a complex, multistage probability sampling design to ensure that the data is nationally representative (Paulose‐Ram et al. 2017; Centers for Disease Control and Prevention, n.d.‐a). NHANES encompasses a wide‐ranging database that reflects the health and nutritional status of a diverse, noninstitutionalized civilian population across various racial and ethnic backgrounds. This resource facilitates extensive research into the relationships between lifestyle, dietary habits, and health outcomes through physical examinations, laboratory tests, and interview data (Centers for Disease Control and Prevention, n.d.‐b). The dietary data collected via the 24‐h dietary recall method allow researchers to analyze associations between nutrient intake and conditions such as obesity, malnutrition, and diet‐related diseases, thereby providing a scientific basis for public health policy. This study utilized NHANES data from 2005 to 2018, incorporating 24‐h dietary recall surveys (Day 1 and Day 2 data), physical examination data (e.g., height and weight), and laboratory results (e.g., serum creatinine). These datasets were employed to estimate the eGFR and to investigate potential associations between tea consumption and the risk of CKD.

### 2.2 Study Population

#### 2.2.1 Participants

**FIGURE 1:**
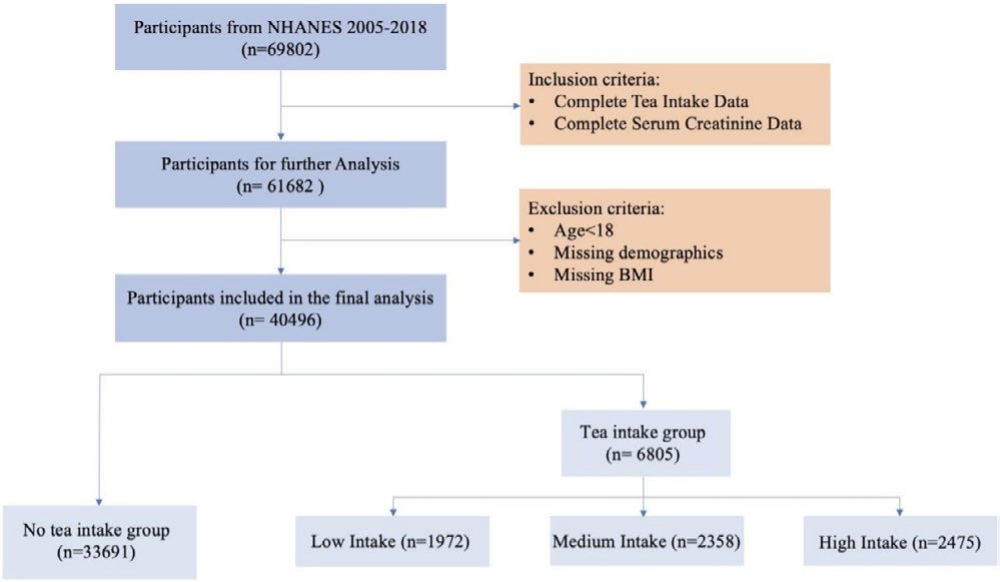
Flowchart of participant selection and inclusion.

This study included adult participants aged 18 years or older who provided complete dietary recall data and had valid serum creatinine test results. To ensure data completeness and the reliability of the study findings, participants with missing dietary recall data or serum creatinine measurements were excluded. To minimize the potential for reverse causality, participants diagnosed with CKD were excluded based on NHANES self‐reported and diagnostic data. This exclusion is essential, as these individuals may have altered their dietary habits, including tea consumption, due to known kidney dysfunction, which could bias causal inference. Additionally, participants with other severe underlying conditions were excluded, including but not limited to active malignancies, severe cardiovascular diseases (e.g., heart failure, acute myocardial infarction), and chronic liver diseases. Such conditions could interfere with the study results by affecting renal metabolism or individual lifestyle patterns. After applying these criteria, a total of 40,496 eligible adult participants were included in the analysis. Participants were further classified into high, medium, low, and no‐tea consumption groups based on their tea intake levels and the timing of consumption, as illustrated in Figure 1.

#### 2.2.2 Exposure Variables and Grouping

Tea consumption data were obtained from the NHANES 24‐h dietary recall survey, utilizing specific food codes to accurately identify tea beverages, including green tea, black tea, and oolong tea. Participants self‐reported the types and quantities of tea consumed, which were subsequently verified through quality control procedures to ensure data accuracy and consistency. To explore the potential association of tea consumption timing on health, this study categorized tea intake into four time periods: dawn to noon (6:00–12:00), noon to afternoon (12:00–18:00), afternoon to night (18:00–24:00), and night to dawn (0:00–6:00). Notably, the inclusion of the night‐to‐dawn time period accounted for individuals working night shifts (e.g., doctors, drivers, security personnel) who may consume tea at night to maintain alertness. Since nighttime tea consumption could be associated with unique responses on kidney function, it was analyzed as a distinct time period to investigate its differential associations on renal function compared to other time periods. This categorization considered potential variations in metabolic and physiological responses to tea consumption across different times of the day. Additionally, participants were divided into three groups based on tertiles of their average daily tea intake: low intake (≤ 178 g/day), medium intake (179–360 g/day), and high intake (> 360 g/day). Tea consumption was calculated from two 24‐h dietary recalls in the NHANES database and refers to the actual amount of brewed tea consumed (in grams, approximately equivalent to milliliters), rather than the weight of dry tea leaves used for brewing. Participants who did not report any tea consumption during any time period were classified as the no‐tea group, serving as the control group for evaluating the associations between tea consumption, kidney function indicators, and CKD risk. This grouping method was designed to clearly delineate the associations of varying tea intake levels and timing on study outcomes, thereby providing a basis for further analysis of the relationship between tea consumption and kidney function.

#### 2.2.3 Covariates

Demographic data were collected from NHANES household interviews, encompassing basic participant information such as age, gender, and race. These variables provide essential background information for analyzing the associations between tea consumption and kidney function across various population groups. Body mass index (BMI) was calculated using height and weight measurements recorded during physical examinations, employing the formula BMI = kg/m^2^. As an indicator of body fatness, BMI is closely linked to the risk of CKD and is an important confounding factor that necessitates adjustment in the study. Smoking status was classified based on questionnaire responses, distinguishing between current smokers and nonsmokers. This classification enabled the study to evaluate the potential impact of smoking as a lifestyle factor on the outcomes. Serum creatinine levels, measured using standardized laboratory methods, served as the primary indicator for estimating the eGFR. The accuracy and consistency of these measurements were maintained through rigorous quality control procedures. The eGFR was calculated using the established CKD‐EPI (Chronic Kidney Disease Epidemiology Collaboration) equation, which adjusts for gender, race, and age (Kidney Disease: Improving Global Outcomes (KDIGO) CKD Work Group 2024). The formula is as follows:

$$\text{eGFR}=141\times \min{\left(\frac{\kappa }{\mathrm{Scr}},1\right)}^{\alpha }\times \max{\left(\frac{\kappa }{\mathrm{Scr}},1\right)}^{-1.209}\times 0.993^{\mathrm{Age}}\times 1.018$$

The parameters in the formula are defined as follows: *κ* = 0.7 for females and *κ* = 0.9 for males; *α* = −0.329 for females and *α* = −0.411 for males; Scr represents serum creatinine (unit: mg/dL), and Age refers to the participant's age. This formula is widely utilized for diagnosing and staging CKD. Its adjustment factors allow for more accurate estimations of eGFR that reflect renal function across different genders and racial groups. By incorporating standardized laboratory data alongside the CKD‐EPI formula, this study systematically evaluated participants' kidney function, thereby providing a robust scientific foundation for analyzing the relationship between tea consumption and eGFR.

### 2.3 Statistical Analysis

Initially, descriptive statistical analyses were performed to delineate the key demographic characteristics and health indicators across various tea consumption groups. Continuous variables, such as age, BMI, and eGFR, were summarized using medians and interquartile ranges (median [IQR]) to more accurately represent the data distribution. Categorical variables, including gender, race, and smoking status, were summarized as sample counts and percentages (*n* [%]). To compare differences among tea consumption groups, analysis of variance (ANOVA) was employed for continuous variables, while the chi‐square test was utilized for categorical variables. To mitigate the risk of false‐positive results arising from multiple comparisons, corrections for multiple comparisons were implemented across all group analyses.

To assess the independent associations of tea consumption levels and timing on eGFR, multivariate linear regression models were developed, with the no‐tea group designated as the reference group. Potential confounders, including age, gender, race, BMI, and smoking status, were included in the models. The outcomes of the regression analyses were presented as regression coefficients accompanied by 95% confidence intervals, thereby quantifying the relationship of varying tea consumption levels on eGFR and evaluating their statistical significance.

In the analysis of interactive associations, interaction terms were incorporated into the regression models to investigate the relationship between tea consumption levels and timing. The significance of these interaction associations was assessed using *p* values (*p* < 0.05), which helped determine whether the association of tea consumption levels on eGFR varied across different time periods. Furthermore, we performed stratified analyses by age and tea type, constructing separate regression models to assess the associations of tea consumption timing and intake levels with eGFR, thereby revealing potential differences across subgroups. The results from these stratified regression analyses were presented as regression coefficients accompanied by 95% confidence intervals, facilitating the identification of potential subgroup‐specific differences.

The NHANES data are derived from a complex, multistage probability sampling design, necessitating the application of weights to ensure national representativeness in the analyses. This study meticulously accounted for the sampling weights in its statistical analyses, utilizing appropriate software for weighted analysis. Both descriptive statistics and regression analyses incorporated sampling weights to generate nationally representative estimates while correcting for sampling bias. All statistical tests were two‐sided, with a significance threshold established at *p* < 0.05. Data processing and analyses were performed using R (version 4.2.2).

## 3 Results

### 3.1 Baseline Analysis

Participants were categorized into low, medium, and high tea consumption groups, as well as a no‐tea group, based on their tea intake levels. The demographic characteristics of each group were compared, and the results are summarized in Table 1.

**TABLE 1:** Comparison of demographic characteristics across tea consumption groups.

Characteristic	Low intake group	Medium intake group	High intake group	No‐tea intake group	*p*
*N*	1972	2358	2475	33,691	
Age (years)	53.11 (39.54, 67.03)	52.28 (38.26, 66.43)	54.02 (41.17, 68.37)	47.37 (30.92, 63.49)	< 0.001
BMI (kg/m^2^)	27.86 (23.52, 32.48)	28.04 (24.05, 33.14)	28.27 (24.16, 33.37)	29.44 (24.82, 34.63)	< 0.001
eGFR (mL/min/1.73 m^2^)	104.76 (85.49, 122.21)	105.95 (86.78, 123.57)	104.41 (85.29, 121.48)	109.34 (89.09, 127.75)	< 0.001
Gender					
Male (%)	712 (36.11%)	962 (40.8%)	1105 (44.65%)	15,462 (50.4%)	< 0.001
Female (%)	1260 (63.89%)	1396 (59.2%)	1370 (55.35%)	15,214 (49.6%)	
Race					
White (%)	311 (15.77%)	295 (12.51%)	261 (10.55%)	5203 (16.96%)	< 0.001
Hispanic (%)	222 (11.26%)	216 (9.16%)	171 (6.91%)	2929 (9.55%)	
African American (%)	635 (32.2%)	957 (40.59%)	1268 (51.23%)	12,823 (41.8%)	
Asian (%)	400 (20.28%)	445 (18.87%)	296 (11.96%)	7107 (23.17%)	
Other (%)	404 (20.49%)	445 (18.87%)	479 (19.35%)	2614 (8.52%)	
Smoking status					
Smoker (%)	632 (32.05%)	858 (36.39%)	1008 (40.73%)	13,392 (43.66%)	< 0.001
Nonsmoker (%)	1338 (67.85%)	1499 (63.57%)	1466 (59.23%)	17,270 (56.3%)	
Time of tea consumption					
Dawn to noon (%)	722 (36.61%)	1059 (44.91%)	1602 (64.73%)		< 0.001
Noon to afternoon (%)	574 (29.11%)	920 (39.02%)	1468 (59.31%)		
Afternoon to night (%)	692 (35.09%)	904 (38.34%)	1267 (51.19%)		
Night to dawn (%)	41 (2.08%)	67 (2.84%)	134 (5.41%)		

The eGFR revealed significant differences among the groups (*p* < 0.001). The no‐tea group exhibited the highest median eGFR, at 109.34 (IQR: 89.09, 127.75) mL/min/1.73m^2^, while the high tea consumption group displayed the lowest median eGFR, at 104.41 (IQR: 85.29, 121.48) mL/min/1.73 m^2^. In terms of demographic characteristics, age significantly differed across the groups (*p* < 0.001). The high tea consumption group had the highest median age, 54.02 (IQR: 41.17, 68.37) years, whereas the no‐tea group had the lowest median age, 47.37 (IQR: 30.92, 63.49) years, indicating that participants in the high‐consumption group were relatively older. Gender distribution also demonstrated significant differences among the groups (*p* < 0.001). The high tea consumption group had a higher proportion of females (55.35%), while the no‐tea group comprised a majority of males (50.4%). Both the low and medium tea consumption groups also had a higher proportion of females, at 63.89% and 59.2%, respectively. Regarding racial composition, significant differences were observed across the groups (*p* < 0.001). The high tea consumption group had the highest proportion of African American participants (51.23%), whereas the no‐tea group had a higher proportion of White participants (16.96%). Hispanic and Asian participants were more prevalent in the low and medium tea consumption groups, accounting for 11.26% and 20.28%, respectively. The proportion of other racial groups was relatively low across all groups.

Significant differences in BMI were observed across the tea consumption groups (*p* < 0.001). The median BMI in the high‐consumption group was 28.27 (IQR: 24.16, 33.37), compared to 27.86 (IQR: 23.52, 32.48) in the low‐consumption group and 29.44 (IQR: 24.82, 34.63) in the no‐tea group. Smoking status also varied significantly among the groups (*p* < 0.001). In the high‐consumption group, 40.73% of participants were smokers, while the proportion was higher in the no‐tea group at 43.66%. In contrast, the low‐ and medium‐consumption groups had a greater proportion of nonsmokers, at 67.85% and 63.57%, respectively.

An analysis of tea consumption timing revealed significant differences in distribution across the groups (*p* < 0.001). Among the high‐consumption group, the highest proportion of participants consumed tea during the dawn‐to‐noon period, at 64.73%, indicating a preference for tea intake early in the day. This was followed by tea consumption from noon to afternoon (59.31%) and from afternoon to night (51.19%). Tea consumption during the night‐to‐dawn period was markedly lower, at only 5.41%, reflecting a tendency among participants in the high‐consumption group to favor daytime tea consumption.

The distributions of tea consumption timing in the low‐ and medium‐consumption groups differed from those in the high‐consumption group. While these groups also demonstrated a preference for daytime tea consumption, the proportions of morning and afternoon tea drinkers were notably lower, suggesting variations in tea‐drinking preferences among these groups. Overall, participants in the high‐consumption group were more likely to consume tea in the morning and during the day, with lower proportions consuming tea at night. These patterns warrant further investigation into their potential associations on kidney function.

### 3.2 The Impact of Tea Consumption Timing on eGFR: Multivariate Regression Analysis

Although descriptive statistics indicated that the no‐tea group had the highest mean eGFR, the results from multivariate regression analysis, adjusted for confounding factors, revealed a significant association between tea consumption at any time of the day and increased eGFR (Table 2). The observed differences may be attributed to the influence of confounding variables, such as age, BMI, and racial composition, on the descriptive statistics. Specifically, tea consumption during the dawn‐to‐noon period exhibited the strongest association with higher eGFR levels, with an average increase of 3.18 compared to the no‐tea group (*p* < 0.001), demonstrating a significant advantage. Tea consumption during the noon‐to‐afternoon and afternoon‐to‐night periods was associated with increases in eGFR of 1.12 and 0.71, respectively, both of which were statistically significant (*p* < 0.001). Furthermore, tea consumption during the night‐to‐dawn period showed a smaller increase in eGFR of 0.44, which was also statistically significant (*p* < 0.001). These findings indicate that tea consumption, irrespective of timing, is significantly associated with higher eGFR levels compared to no‐tea consumption. Notably, the association was most pronounced during the dawn‐to‐noon period, suggesting that morning tea consumption may be particularly favorable in relation to kidney function.

**TABLE 2:** Analysis of the impact of tea consumption timing and covariates on eGFR levels in the multivariate regression model.

Variable	Regression coefficient (estimate)	Standard error	*t*‐value	*p*
Intercept	136.71	0.30	455.08	< 0.001
Dawn to noon	3.18	0.29	10.94	< 0.001
Noon to afternoon	1.12	0.12	9.36	< 0.001
Afternoon to night	0.71	0.12	5.87	< 0.001
Night to dawn	0.44	0.12	3.51	0.0004

### 3.3 Comprehensive Assessment of Tea Consumption Timing and Intake Levels on eGFR

This study employed linear regression models to assess the associations of tea consumption levels at various times of the day and their interactive associations on eGFR (Table 3). The results indicated significant differences in eGFR levels associated with the timing and levels of tea consumption. High tea consumption during the dawn‐to‐noon and noon‐to‐afternoon periods demonstrated nearly significant positive associations on eGFR, with regression coefficients of 1.6474 (*p* = 0.0390) and 1.7664 (*p* = 0.0261), respectively. These findings suggest that high intake during these periods may be associated with higher eGFR levels. Moderate tea consumption in the afternoon‐to‐night period also exhibited a slight positive association, with a regression coefficient of 1.2905 (*p* = 0.0075), indicating that moderate tea consumption during this timeframe may be associated with higher eGFR levels. Conversely, during the night‐to‐dawn period, high tea consumption yielded a regression coefficient of 1.9238 (*p* = 0.4090), which was not statistically significant, suggesting no clear trend in eGFR changes associated with high intake at night. However, moderate tea consumption during this period was linked to a regression coefficient of −3.5468 (*p* = 0.0011), indicating a potential negative association with eGFR. It is crucial to recognize that this negative association may be influenced by sample characteristics or unadjusted confounding factors, and thus, it is insufficient to definitively conclude that nighttime tea consumption is detrimental to kidney function. These findings underscore the complex relationship between tea consumption timing, intake levels, and kidney function, warranting further investigation to validate these results and explore the underlying mechanisms. Additionally, the model revealed interaction associations across different time periods. A significant positive interaction association was observed for moderate tea consumption during the dawn‐to‐noon and noon‐to‐afternoon periods, with a regression coefficient of 4.2521 (*p* = 0.0186). This finding suggests that moderate tea consumption during these two time frames may be more strongly associated with higher eGFR levels.

**TABLE 3:** Multivariate regression analysis of tea consumption timing and intake levels on eGFR.

Variable	Estimate	Standard error	*t*‐value	*p*
Intercept	138.0451	1.71857	80.326	< 0.001
Dawn to noon				
Low intake	0.58141	0.84825	0.685	0.4931
Medium intake	−0.19687	0.84880	−0.232	0.8166
High intake	1.6474	0.87225	1.889	0.0390
Noon to afternoon				
Low intake	0.25437	0.90404	0.281	0.7784
Medium intake	0.5144	0.85956	0.598	0.5496
High intake	1.76639	0.92447	1.911	0.0261
Afternoon to night				
Low intake	0.77431	0.70485	1.099	0.2720
Medium intake	1.29054	0.70584	1.828	0.0075
High intake	0.78233	0.70299	1.113	0.2658
Night to dawn				
Low intake	1.29847	2.21120	0.587	0.5571
Medium intake	−3.54683	2.22582	−1.593	0.0011
High intake	1.92377	2.33003	0.826	0.4090
Age	−0.93384	0.01195	−78.122	< 0.001
Gender	19.68448	0.43135	45.634	< 0.001
Race	−1.87614	0.17354	−10.811	< 0.001
BMI	−0.22911	0.03292	−6.959	< 0.001
Smoking status	−1.92607	0.41948	−4.592	< 0.001

Overall, the study found that moderate tea consumption during the morning and noon periods was significantly positively associated with higher eGFR levels of kidney function, while moderate intake at night may be associated with negative associations. Future research should further validate these findings and explore the specific mechanisms by which the active components in tea are associated with changes in kidney function at different times of consumption, thereby providing a foundation for developing evidence‐based tea consumption guidelines.

### 3.4 Age‐Stratified Analysis Results

In this section, a stratified regression analysis was conducted by dividing the sample into three age groups: young adults (18–39 years), middle‐aged adults (40–59 years), and older adults (60 years and above). This analysis evaluated the association of tea consumption timing on eGFR levels within each group. The results indicated significant differences in the relationship between tea consumption timing and eGFR levels across the age groups (Table 4). Additionally, a stratified analysis based on gender was performed; however, the results did not reveal significant differences. This suggests that gender may not be a key moderating factor in the associations of tea consumption timing on kidney function.

**TABLE 4:** Age‐stratified analysis of the impact of tea consumption timing on eGFR levels.

Analysis variable	Young adults (18–39 years)	Middle‐aged adults (40–59 years)	Older adults (≥ 60 years)
Model fit indicators			
*R*‐squared	0.5907	0.367	0.305
Residual standard error	14.95	14.21	17.44
Dawn to noon			
Regression coefficient	−1.64	3.79	6.17
*p*‐value	0.00769	< 0.001	< 0.001
Noon to afternoon			
Regression coefficient	—	1.66	3.28
*p*‐value	> 0.05	< 0.001	< 0.001
Afternoon to night			
Regression coefficient	—	1.08	3.32
*p*‐value	> 0.05	< 0.001	< 0.001
Night to dawn			
Regression coefficient	−0.52	0.68	3.22
*p*‐value	0.01756	0.0004	< 0.001

In the young adult group (ages 18–39 years), tea consumption during the night‐to‐dawn and dawn‐to‐noon periods was found to have a significant negative association on eGFR levels, with regression coefficients of −1.64 and −0.52 (*p* = 0.00769 and *p* = 0.01756, respectively), indicating a slight downward trend. Conversely, tea consumption during the afternoon‐to‐night and noon‐to‐afternoon periods did not exhibit significant associations on eGFR levels (*p* > 0.05). These findings suggest that tea consumption during the night‐to‐dawn and dawn‐to‐noon periods may have a mild adverse association on kidney function in young adults, while tea consumption at other times of the day appears to have minimal or no discernible association.

In the middle‐aged group (40–59 years), tea consumption across all time periods was associated with a significant increase in eGFR levels. The most pronounced association was observed for tea consumption during the dawn‐to‐noon period (regression coefficient: 3.79, *p* < 0.001); followed by the noon‐to‐afternoon period (regression coefficient: 1.66, *p* < 0.001), the afternoon‐to‐night period (regression coefficient: 1.08, *p* < 0.001), and the night‐to‐dawn period (regression coefficient: 0.68, *p* = 0.0004). These findings suggest that tea consumption at any time of the day may be associated with higher eGFR levels in middle‐aged adults, with particularly pronounced associations during the dawn‐to‐noon and noon‐to‐afternoon periods.

In the older adult group (aged 60 years and above), tea consumption across all time periods was significantly associated with increased levels of eGFR. The most pronounced association was observed during the dawn‐to‐noon period (regression coefficient: 6.17, *p* < 0.001), followed by the afternoon‐to‐night period (regression coefficient: 3.32, *p* < 0.001), the noon‐to‐afternoon period (regression coefficient: 3.28, *p* < 0.001), and the night‐to‐dawn period (regression coefficient: 3.22, *p* < 0.001). These findings suggest that tea consumption at any time of day may be associated with higher eGFR levels in older adults, with the most significant associations noted during the dawn‐to‐noon period.

In summary, stratified analysis revealed significant differences in the associations of tea consumption timing on eGFR levels across various age groups. Among young adults, tea consumption during the night‐to‐dawn and afternoon‐to‐night periods was associated with a slight decrease in eGFR levels, potentially reflecting the influence of tea‐drinking habits and lifestyle factors prevalent in this demographic. In contrast, both middle‐aged and older adults exhibited significant increases in eGFR levels across all periods of tea consumption, with the most pronounced associations observed during the dawn‐to‐noon and noon‐to‐afternoon intervals. These findings may indicate a greater preference for daytime tea consumption among middle‐aged and older adults, which is linked to positive associations on kidney function.

### 3.5 Subgroup Analysis by Tea Type

In the stratified analysis by tea type, intake level, and time of consumption, distinct patterns of associations with eGFR were observed (Table 5).

**TABLE 5:** Associations between tea consumption timing, intake levels, and eGFR across different tea types.

Variable	Low		Medium		High	
	*β* (95% CI)	*p*	*β* (95% CI)	*p*	*β* (95% CI)	*p*
Black tea						
Dawn to noon	−0.52 (−1.58 to 0.54)	0.334	0.49 (−0.45 to 1.43)	0.309	2.82 (1.94 to 3.71)	< 0.001
Noon to afternoon	0.95 (−0.08 to 1.98)	0.071	1.04 (0.10 to 1.98)	0.030	3.14 (2.24 to 4.03)	< 0.001
Afternoon to night	−1.01 (−2.07 to 0.04)	0.060	0.05 (−0.90 to 1.00)	0.914	2.91 (2.02 to 3.80)	< 0.001
Night to dawn	0.02 (−3.35 to 3.39)	0.990	0.14 (−2.28 to 2.56)	0.912	2.29 (0.49 to 4.09)	0.013
Green tea						
Dawn to noon	−0.25 (−2.51 to 2.02)	0.832	0.13 (−1.88 to 2.15)	0.896	1.61 (−0.27 to 3.49)	0.094
Noon to afternoon	−1.24 (−3.46 to 0.98)	0.274	−0.44 (−2.45 to 1.58)	0.671	1.69 (−0.23 to 3.62)	0.084
Afternoon to night	0.05 (−2.22 to 2.33)	0.964	0.01 (−2.03 to 2.04)	0.996	1.50 (−0.44 to 3.44)	0.130
Night to dawn	−5.36 (−13.27 to 2.54)	0.183	−1.22 (−7.05 to 4.62)	0.683	5.68 (1.91 to 9.45)	0.003
Other tea						
Dawn to noon	−0.46 (−2.61 to 1.70)	0.679	−0.92 (−2.96 to 1.13)	0.379	0.94 (−0.95 to 2.83)	0.332
Noon to afternoon	−2.81 (−5.22 to −0.39)	0.023	−0.58 (−2.64 to 1.47)	0.580	0.17 (−1.63 to 1.98)	0.850
Afternoon to night	2.47 (0.30 to 4.64)	0.026	2.99 (0.98 to 5.00)	0.004	1.76 (−0.14 to 3.66)	0.070
Night to dawn	−2.13 (−8.58 to 4.32)	0.518	−5.51 (−11.98 to 0.95)	0.095	−8.22 (−12.44 to −4.00)	< 0.001

For black tea, higher intake levels were consistently associated with higher eGFR across different time periods. Specifically, high intake during dawn to noon (*β* = 2.82, 95% CI: 1.94–3.71, *p* < 0.001); noon to afternoon (*β* = 3.14, 95% CI: 2.24–4.03, *p* < 0.001); afternoon to night (*β* = 2.91, 95% CI: 2.02–3.80, *p* < 0.001); and night to dawn (*β* = 2.29, 95% CI: 0.49–4.09, *p* = 0.013) showed significant positive associations. Medium intake during noon to afternoon (*β* = 1.04, 95% CI: 0.10–1.98, *p* = 0.030) was also positively associated with eGFR. No significant associations were observed for low intake levels.

For green tea, a significant positive association was found only for high intake during night to dawn (*β* = 5.68, 95% CI: 1.91–9.45, *p* = 0.003). No significant associations were observed for low or medium intake levels across other time periods.

For other tea types, low (*β* = 2.47, 95% CI: 0.30–4.64, *p* = 0.026) and medium (*β* = 2.99, 95% CI: 0.98–5.00, *p* = 0.004) intake levels during afternoon to night were positively associated with eGFR. However, a significant negative association was observed for low intake during noon to afternoon (*β* = −2.81, 95% CI: −5.22 to −0.39, *p* = 0.023). Additionally, high intake during night to dawn was negatively associated with eGFR (*β* = −8.22, 95% CI: −12.44 to −4.00, *p* < 0.001).

Taken together, these findings suggest complex and heterogeneous associations between tea consumption timing, intake levels, and eGFR across different tea types.

## 4 Discussion

This study, utilizing data from the NHANES, examined the potential association of tea consumption timing on changes in eGFR and the risk of CKD. The results indicated that tea consumption, irrespective of the timing, was linked to elevated eGFR levels, with the most significant associations noted during the dawn‐to‐noon period. These findings offer important evidence for refining tea consumption strategies related to kidney health.

The study results demonstrated that tea consumption was significantly associated with higher eGFR levels across all time periods, although its associations exhibited a clear time dependency. While research on the timing of tea consumption is limited, potential advantages during specific periods can be inferred from tea's health properties and the body's natural circadian rhythms. Participants who consumed tea during the dawn‐to‐noon period showed significantly higher eGFR levels compared to those who consumed it at other times. This may be attributed to the increased metabolic activity of the kidneys in the morning, along with the enhanced antioxidant properties of tea polyphenols during this period. The body's metabolic processes are notably influenced by circadian rhythms, with the morning period representing a metabolic peak. During this time, the kidneys' detoxification and metabolic efficiency are at their highest, which may facilitate more effective absorption of tea's polyphenolic compounds. These active substances may help protect the glomeruli and renal tubules from damage by scavenging reactive oxygen species and reducing oxidative stress (Tang, Zhao, et al. 2019; Braud et al. 2015). Furthermore, the anti‐inflammatory properties of tea polyphenols inhibit the secretion of pro‐inflammatory cytokines such as IL‐6 and TNF‐α, which may mitigate the progression of chronic renal inflammation. This protective mechanism may be more readily engaged during the heightened metabolic activity of the morning (Khan and Mukhtar 2018; Wan et al. 2021). Additionally, the digestive system is relatively active in the early morning, which may enhance the bioavailability of antioxidants in tea. For example, catechins are absorbed more efficiently on an empty stomach, resulting in a higher concentration of these protective compounds in the bloodstream, which may enhance their health‐related properties (Wu et al. 2023). Daytime tea consumption also contributes to the stabilization of blood circulation and renal perfusion, which may be associated with a higher GFR (Haghighatdoost et al. 2021). Morning tea consumption provides hydration after an overnight fast, which may promote the elimination of toxins and may optimize overall metabolic health. This detoxification process is particularly crucial in the morning, when the body is primed to absorb nutrients and excrete waste (Qin et al. 2024). Regular consumption of morning tea has also been associated with a range of long‐term health benefits, including reduced risks of cardiovascular disease and certain types of cancer (Chung et al. 2020; Cao et al. 2019; Krull Abe and Inoue 2020). These protective associations likely stem from the cumulative actions of antioxidants and other beneficial compounds, which include lowering cholesterol levels and improving vascular function (Chen et al. 2019). Moreover, morning tea rituals offer psychological and social benefits. As a practice that fosters mindfulness and relaxation, the consumption of morning tea may help set a positive tone for the day ahead. This psychological boost not only may enhance daily well‐being but also may contribute to overall health (Bryan et al. 2012).

In contrast, tea consumption during the night‐to‐dawn period demonstrated weaker associations with higher eGFR levels, with high intake potentially being associated with negative associations on kidney function. This phenomenon may be attributed to the decline in renal detoxification rhythms and metabolic activity during the night. Nighttime tea consumption may disrupt the kidneys' natural detoxification rhythms, while the caffeine in tea could further interfere with sleep patterns, leading to insufficient rest (Derbyshire et al. 2023; Dewer et al. 2023). Sleep is critical for overall health, including the maintenance of kidney function, as it represents a key period for bodily repair and regeneration. Chronic sleep deprivation has been associated with a range of health issues, including hypertension and diabetes, both of which are significant risk factors for CKD (Xu et al. 2022; Hoopes et al. 2022; Jiang et al. 2023). Additionally, caffeine is a known diuretic. While moderate caffeine intake can promote urine production and facilitate toxin excretion, excessive intake may lead to increased urine output, potentially resulting in dehydration. Dehydration poses a significant threat to kidney function, as it reduces renal blood flow, impairs the kidneys' ability to effectively filter waste, and may even increase the risk of acute kidney injury (Chandramohan et al. 2023). This issue may be exacerbated by late‐night consumption of high doses of caffeine. If fluid intake is insufficient to compensate for excessive fluid loss, dehydration can worsen (Shekarforoush and Fardaee 2020). Furthermore, nighttime tea consumption may overactivate the sympathetic nervous system, increasing the burden on the kidneys and diminishing the protective associations of tea (Patel et al. 2022). The type of tea consumed at night is also important to consider; for instance, black tea contains higher levels of oxalates compared to other varieties (Hashiguchi et al. 2023; Chiang et al. 2022). Oxalates are significant contributors to kidney stone formation, particularly in individuals predisposed to stone development (Raja et al. 2023). Excessive consumption of black tea may elevate urinary oxalate levels, thereby increasing the risk of calcium oxalate stones and potentially compromising kidney function. Consequently, individuals who consume large amounts of black tea at night may inadvertently heighten their risk of kidney stone formation, leading to additional complications. Moreover, those who drink tea late at night often experience considerable work or life stress, which can be associated with adverse health outcomes through various pathways. Prolonged states of high stress may trigger chronic inflammation and oxidative stress, which may impair kidney function and exacerbate the risks of hypertension and metabolic disorders, ultimately increasing the likelihood of CKD (Huang et al. 2023).

Stratified analysis further revealed the differential association of tea consumption timing on eGFR across various age groups. In the young adult cohort, tea consumption during the night‐to‐dawn period was associated with a slight negative association on kidney function, potentially linked to sleep disturbances caused by nighttime tea intake (Hoopes et al. 2022). Sleep deprivation can disrupt the kidneys' repair and metabolic rhythms, which may adversely affect renal function in younger individuals (Bo et al. 2019; Cheungpasitporn et al. 2017). In contrast, both middle‐aged and older adults exhibited significant associations with higher eGFR levels with tea consumption across all time periods, with the most pronounced associations observed during the dawn‐to‐noon period. This phenomenon may be attributed to the slower metabolic rate in these age groups, coupled with the cumulative protective associations of tea's active compounds. Notably, results from the older adult group indicated that the dawn‐to‐noon period yielded the greatest associations, suggesting that this demographic may derive enhanced associations from morning tea consumption. Given the elevated risk of CKD in older adults, strategically timing tea consumption may be associated with better kidney function preservation. Optimizing tea‐drinking habits, particularly through moderate consumption in the morning, not only may maximize the associations of tea's active components but also may offer a simple and effective intervention strategy for kidney function protection in older adults. The findings from stratified analysis may reflect variations in dietary and lifestyle habits across different age groups. Younger individuals, influenced by academic, work, or social activities, often display irregular dietary and lifestyle patterns. For example, nighttime tea consumption may serve as a strategy to stay awake or compensate for insufficient sleep (Calamaro et al. 2009). While this behavior is intended to combat fatigue, it may disrupt metabolic health and overall quality of life. Caffeinated beverages, such as tea, may enhance alertness and prolong wakefulness but may interfere with normal sleep patterns, resulting in inadequate sleep duration and poor sleep quality. Research indicates that sleep deprivation is common among young individuals, with approximately 29.7% of those aged 18–24 reporting short sleep durations, which may adversely affect their health and daily functioning (Rubin et al. 2023). Such irregular lifestyles may undermine the overall protective effects of tea consumption and even introduce metabolic stress. In contrast, middle‐aged and older adults tend to be more health‐conscious, often maintaining regular schedules and dietary habits. They may deliberately incorporate tea consumption into their health management routines (Acosta et al. 2021; Gao et al. 2022; Xu et al. 2018). This increased health awareness likely enhances the positive associations of tea on kidney function, particularly when consumed at optimal times, such as in the morning or afternoon. These differences highlight the importance of considering age‐related lifestyle factors when evaluating the health associations of tea consumption.

Our study revealed distinct associations between different tea types and kidney function, suggesting that not all teas exert similar effects on eGFR. Black tea showed generally positive associations with higher eGFR at higher intake levels and across various consumption periods. This could be explained by its unique polyphenols, such as theaflavins and thearubigins, known for their antioxidant and anti‐inflammatory properties, which may support vascular health and renal function (Tang, Meng, et al. 2019; Vazhappilly et al. 2019; Koch 2020; Guerreiro et al. 2022). In contrast, the associations observed for green tea were more variable and less consistent. Although green tea is rich in catechins with recognized health benefits, these effects might be influenced by differences in brewing strength, preparation methods, or individual metabolic responses (Xing et al. 2019; Jin et al. 2019; Sharpe et al. 2016). Additionally, cultural and regional consumption patterns could further modulate these associations, potentially diluting the protective impact observed in controlled experimental studies. For other tea types, including herbal and instant teas, the patterns were more heterogeneous, with some intake levels even showing potential adverse associations with eGFR. These teas often contain additives, sweeteners, or varying caffeine content, which may affect kidney function differently (Rebholz et al. 2019; Lin and Curhan 2011). Particularly, consuming certain teas at night could disrupt circadian rhythms and metabolic balance, potentially contributing to negative renal outcomes. Overall, these findings highlight the need to consider tea type and consumption patterns when evaluating tea's impact on kidney health. Personalized recommendations that take into account individual preferences and tea types may be more effective than generalized advice. Further longitudinal and mechanistic studies are needed to clarify these relationships and inform tailored dietary guidelines.

This study offers valuable insights into dietary intervention strategies that may be associated with better kidney function. It is recommended that tea consumption be concentrated in the morning and daytime to maximize the health associations, while excessive intake during the nighttime should be avoided to mitigate potential adverse associations. This guidance may not only be beneficial for individual health management but also provides a scientific foundation for the development of public health policies.

Although this study revealed significant associations of tea consumption timing on eGFR, several limitations warrant consideration. The tea consumption data in the NHANES database relied primarily on participants' self‐reports, which may introduce recall bias. Furthermore, as a cross‐sectional study, this research cannot establish causal relationships between tea consumption timing and kidney function. Future longitudinal studies are necessary to further validate these associations. Additionally, the mechanisms underlying the absorption and metabolism of tea's active components during different time periods remain unclear. Experimental studies should be conducted to explore the specific regulatory roles of tea's active components on renal metabolism, thereby providing stronger theoretical support for scientific recommendations regarding tea consumption.

## 5 Conclusion

In summary, this study is the first to systematically evaluate the association of tea consumption timing on kidney function. The results reveal that consuming tea in the morning and during the daytime is significantly associated with higher eGFR levels, while high intake during nighttime may have negative associations on kidney function. These findings provide a novel perspective for optimizing tea‐drinking habits and underscore the relationship between tea consumption timing and health associations. Future research aimed at validating and refining these results will contribute to personalized dietary intervention strategies for better kidney function preservation and the prevention of CKD.

## Author Contributions

**Xuetong Tang:** conceptualization (lead), data curation (lead), formal analysis (lead), investigation (lead), methodology (lead), project administration (lead), software (lead), validation (lead), visualization (lead), writing – original draft (lead), writing – review and editing (lead). **Jiankui Guo:** writing – review and editing (equal). **Wen Hu:** supervision (equal). **Yuan Liu:** funding acquisition (equal), supervision (equal), writing – review and editing (equal). **Yunying Shi:** supervision (supporting).

## Conflicts of Interest

The authors declare no conflicts of interest.

## Data Availability

This study utilized data from the NHANES, covering the 2005–2018 cycles. The NHANES datasets are publicly available and can be accessed at https://www.cdc.gov/nchs/nhanes/index.htm. Detailed variable documentation, data dictionaries, and survey methodologies are provided on the NHANES website. All analyses in this study were conducted in accordance with NHANES guidelines and ethical standards for secondary data use.
